# Genome-wide analysis of radish *AHL* gene family and functional verification of *RsAHL14* in tomato

**DOI:** 10.3389/fpls.2024.1401414

**Published:** 2024-05-30

**Authors:** Weifang Chen, Leifu Chen, Lei Cui, Zhixiong Liu, Weiling Yuan

**Affiliations:** Hubei Key Laboratory of Vegetable Germplasm Innovation and Genetic Improvement, Institute of Economic Crops, Hubei Academy of Agricultural Sciences, Wuhan, China

**Keywords:** Radish *(Raphanus sativus*), AHL genes, genome-wide analysis, gene expression, stress response

## Abstract

The *AT-hook motif nuclear localized* (*AHL*) gene family is a highly conserved transcription factors involved in plant growth, development, and stress responses. However, *AHLs* have not been systematically analyzed in radish (*Raphanus sativus*). Therefore, we performed genome-wide identification and expression pattern, gene structure, and function verifications of radish *AHLs*. We identified 52 radish *AHLs* (*RsAHL1–RsAHL52*), which were unevenly distributed across nine chromosomes. Phylogenetic analysis showed that the *RsAHLs* were divided into two clades (A and B) and subdivided into three types (I, II, and III). Collinearity analysis revealed that the 52 *RsAHLs* produced 49 repeat events. Tissue expression profiles revealed differential expression of *RsAHLs* across different tissues, with higher expression observed in flower organs, particularly petals and anthers. qRT-PCR results indicated that *RsAHLs* responded to abscisic acid, methyl jasmonate, and abiotic stress (low and high temperatures and drought). Additionally, *RsAHL14* induced a dwarf phenotype in tomato plants, and *RsAHL14*-overexpression tomato plants presented significantly decreased expression levels of the gibberellin (GA) synthetic genes *ent-Copalyl diphosphatase*, *GA3ox-3/-4/-5*, and *GA20ox-1/-2/-3*, but significantly increased expression of the degradation gene *GA2ox-1/-3*. Thus, *RsAHL14* might affect plant growth by regulating GA content. Collectively, our study comprehensively identified *RsAHLs* in radish and provided a reference for further research on these genes.

## Introduction

Plants are subjected to various biotic and abiotic stresses during growth and development and resist these stresses through their own defense mechanisms (Alves et al., 2014). Transcription factors are activated under stress and play a defensive role by regulating the expression of defense-related genes (Chen et al., 2002). In *Arabidopsis*, AP2/EREBP, bZIP/HD-ZIP, Myb, and several zinc finger (ZF) transcription factors are either activated or suppressed under stress conditions (Shinozaki and Yamaguchi-Shinozaki, 2000). Notably, altering the expression levels of certain transcription factors can significantly alter plant stress resistance. For example, the overexpression of *VvNAC17* can improve the sensitivity of *Arabidopsis* to abscisic acid (ABA) and enhance the ability of *Arabidopsis* to resist drought, salt, and freezing stress (Ju et al., 2020).

*AT-hook motif nuclear localized* (*AHL*) genes are transcription factors that were first discovered in the high-mobility group proteins (HMG) of mammals, and they play an important role in chromosome structure assembly and target gene transcription regulation (Goodwin et al., 1973). *AHL* genes are widely present in terrestrial plants. For example, 29 *AHL* genes were identified in *Arabidopsis* (Zhao et al., 2014), 37 in maize (Bishop et al., 2020), 63 in soybean (Wang et al., 2021b), 26 in rice (Kumar et al., 2023), 47 in carrot (Machaj and Grzebelus, 2021), 42 in *Brassica rapa* (Zhang et al., 2023), and 48, 51, and 99 in the three different cotton genomes (Zhao et al., 2020), respectively. AHL proteins contain two conserved domains: the AT-hook motif and the plants and prokaryotes conserved (PPC) domain (Zhao et al., 2013). The AT-hook motif is a small DNA-binding motif that can be divided into two types (I and II). Type I AT-hook contains the conserved sequence Arg-Gly-Arg-Pro, followed by Gly-Ser-Lys-Asn-Lys, while Type II AT-hook contains the conserved sequence Arg-Gly-Arg-Pro-Arg-Lys-Tyr. Both types have a characteristic structure centered on Arg-Gly-Arg-Pro (RGRP) residues (Zhang et al., 2022). This conserved amino acid sequence is necessary for protein-DNA interactions and nuclear localization (Do et al., 2006). The AT-hook motif specifically binds to AT-rich sequences in double-stranded DNA furrows to complete interactions with target genes (Aravind and Landsman, 1998). The PPC domain, also known as DUF296, contains approximately 120 amino acids and is located in the carboxylic acids relative to the AT-hooks (Preston and Hileman, 2012). PPC domains can be divided into Type A (without introns) and Type B (with introns), both of which contain a Gly-Arg-Phe-Glu-Ile-Leu core conserved sequence. Upstream of the conserved sequence, the Type A PPC domain has the Leu-Arg-Ser-His core conserved sequence, while the Type B PPC domain has the Phe-Thr-Pro-His core conserved sequence (Zhang et al., 2022). In plants, the main roles of PPC domains are nuclear localization and protein-protein interactions, suggesting that AHL proteins may be involved in regulating transcriptional activity (Fujimoto et al., 2004).

Based on the AT-hook motif sequence characteristics, sequence similarity, AT-hook motif combination with PPC, and affinity for DNA, AHL proteins can be divided into three categories: Types I, II, and III. Type I AHL proteins have polar amino acids at the C-end of the core sequence, and the second amino acid at the C-end is usually glycine, which has strong affinity. Type I AHL proteins contains one Type I AT-hook motif and Type A PPC domain. The second amino acid at the C-terminus of the Type II AHL proteins core sequence is usually lysine, which has a weak affinity. Type II AHL proteins contain two AT-hook motifs (Type I and Type II) and Type B PPC domain. The fourth conserved amino acid downstream of the C terminal of the Type III AHL proteins is lysine, and its affinity lies between those of Types I and II. Type III AHL proteins contains one Type II AT-hook motif and Type B PPC domain. Furthermore, the *AHL* proteins could be divided into two clades (clade A and clade B), in which Type I AHL proteins belonged to clade A, while Types II and III AHL proteins belonged to clade B (Zhao et al., 2014). Analysis of exon and intron numbers of maize *AHL* genes shows that Types II and III evolved from Type I (Bishop et al., 2020). AHL proteins can not only directly bind DNA but also affect the binding of other transcription factors to DNA, thereby indirectly regulating the transcriptional activity of target genes (Strick and Laemmli, 1995).

*AHL* genes are crucial for plant growth and development, organ building, and stress responses. In *Arabidopsis thaliana*, AHL22 can act as a chromatin remodeling factor to modify the structure of *FLOWERING LOCUS T* (*FT*) chromatin and regulate flowering time (Yun et al., 2012). Overexpression of *AHL20* and *AHL22* decreases the transcription levels of *FT* and delays flowering (Xiao et al., 2009; Yun et al., 2012; Tayengwa et al., 2020). Furthermore, AHL22, AHL27, and AHL29 negatively regulate hypocotyl elongation (Street et al., 2008; Zhao et al., 2013). AHL transcription factors and phytochrome-interacting factors (PIF) competitively bind to PIF target sites, reduce PIF binding to growth-promoting genes, and inhibit the transcriptional activation of these genes, thus repressing leaf petiole elongation (Favero et al., 2020). TEK/AHL16 could negatively regulate the flowering inhibitors *MAF4* and *MAF5* (Xu et al., 2013). During the aging process, *AHL27* could delay the aging of *Arabidopsis* leaves, while *AHL9* showed the opposite phenotype (Lim et al., 2007; Zhou et al., 2022). In plant defense and stress responses, overexpression of *OsAHL1* could improve the drought resistance of rice (Zhou et al., 2016). Further studies showed that the *OsAHL10*, *OsAHL13* and *OsAHL20* were involved in the signaling of drought stress and salt stress (Ambadas et al., 2023). However, overexpression of *AHL20* inhibited the expression of *NHO1* and *FRK1* induced by pathogen-associated molecular patterns (PAMPs) and was sensitive to toxic *Pseudomonas syringae* bacteria, indicating that AHL20 negatively regulates the defense ability of *Arabidopsis* (Lu et al., 2010). In addition, several *AHL* genes have been reported to regulate plant hormone balance, such as gibberellin (Matsushita et al., 2007), cytokinin (Rashotte et al., 2003), and jasmonic acid (Vom Endt et al., 2007).

Radish (*Raphanus sativus*) is an important cruciferous vegetable and one of the most widely cultivated root vegetables, and studies have suggested that radish can be used as an ideal model plant for root crops (Hoang et al., 2020). During radish growth, biotic and abiotic stresses can affect the growth and expansion of taproots, thus affecting radish yield (Hoang et al., 2020; Li et al., 2023). To date, studies on *AHL* genes have mainly focused on *Arabidopsis*, whereas studies on *AHL* genes in radishes are relatively scarce. Therefore, to gain a more comprehensive understanding of the important functions of *AHL* genes in plants, we identified and analyzed the *RsAHL* family in radishes in this study. The gene structure, phylogenetic tree, chromosome location, gene collinearity, conserved motifs, and promoter cis-elements of radish *RsAHL* family members were analyzed. Specific expression patterns of *RsAHL* family were also identified in various tissues, and their response to abiotic and hormone stress was observed. Furthermore, we identified the biological functions of *RsAHL14* overexpression in tomatoes. Therefore, this study lays the foundation for further analysis of the role of *RsAHL* genes in radish growth, development, and stress responses.

## Materials and methods

### Identification of the *RsAHL* family in radish

The radish genome “NAU-LB” (BioProject number: PRJCA011486.) (Xu et al., 2023) was used to identify *RsAHL* family members in radish. Twenty-nine *Arabidopsis* and twenty-six rice AHL family protein sequences were used to construct a Hidden Markov Model (HMM) of known AHL protein family sequences using HMMER 3.0 software. The HMM was generated by computational analysis of known homologous gene sequences and used to predict whether the homologous gene sequences exist in other species. All potential RsAHL family sequences were identified in the radish protein sequences. In contrast, BLASTP (Altschul et al., 1990) (v2.10.1+) was used to compare all radish protein sequences with the obtained *AHL* family reference sequences (e-value=1*10^–5^), and the matched sequences were used as potential *RsAHL* family sequences. After synthesizing these candidate sequences, the PfamScan (Bateman et al., 2004) (v1.6) and Pfam A (Finn et al., 2014) (v33.1) databases were used to annotate the domain of the target sequence, and the sequence containing the PF03479 domain was determined as the final RsAHL protein sequence.

### Phylogenetic tree analysis of the *RsAHL* family

The RsAHL protein family sequences of radish, *Arabidopsis*, and rice were used to construct a neighbor-joining tree. First, MAFFT (v7.427) was used to perform multiple sequence comparisons, and MEGA10 software (Kumar et al., 2008) was used to build the neighbor-joining tree (the bootstrap was set to 1000), and then iTOL v6 (https://itol.embl.de/) was used to annotate the evolutionary tree.

### Chromosome localization analysis of the *RsAHL* family in radish

“NAU-LB” genome sequence and general feature format (GFF) files were used to extract chromosome length and location information of *RsAHL* family members through the TBtools software. Subsequently, further visualization analysis was performed (Chen et al., 2020a).

### Collinearity analysis of *RsAHLs*

Collinearity analysis was performed using MCScanX software (match score, 50; match size, 5; gap penalty, -1; overlap window, 5; e-value, 1e^-5^; max gaps, 25). Segment and tandem duplications caused by gene duplication events were analyzed.

### Gene structure and conserved motif analysis of *RsAHLs*

The CDS and genome sequences of radish *RsAHL* family members were used to analyze the intron-exon structure using the GSDS website (http://gsds.cbi.pku.edu.cn/) for visualization. The MEME website was used to predict the conserved motif of *RsAHLs*, and TBtools software was used to visualize it.

### Promoter cis-element analysis of *RsAHLs*

The upstream 2 kb sequences of the *RsAHLs* promoter were extracted from the “NAU-LB” genome and then submitted to the PlantCARE (https://bioinformatics.psb.ugent.be/webtools/plantcare/html/) database to predict the distribution of cis-elements on the promoter. Subsequently, all cis-elements were classified, and those related to stress and hormone responses were extracted for visual analysis using the TBtools software.

### Plant materials and stress treatments

Radish (*Raphanus sativus*) was cultivated at 25°C with 16 h of light and 8 h of darkness per day. Radish materials with consistent growth were selected for the stress treatment. Radish leaf samples were collected after cold treatment (4°C), high temperature treatment (42°C), PEG6000 treatment and hormone treatment (methyl jasmonate, abscisic acid) at 0 h, 1 h, 3 h, 6 h, 9 h, 12 h and 24 h.

### Genetic transformation

The CDS of *RsAHL14* was amplified using radish cDNA with specific amplification primers (**Supplementary Table 4**) and then cloned into pHELLSGATE8 (Chen et al., 2020b) to generate CaMV35S::AHL14 overexpression vector. This vector was transferred into *Agrobacterium* strain GV3101. Subsequently, genetic transformation was performed using MicroTom tomatoes as the background material. Transgenic plants were detected using PCR, and the primer sequences are shown in **Supplementary Table 4**.

### RNA extraction and real-time fluorescence quantitative PCR

Total RNA was extracted using TRIzol reagent (Vazyme, Nanjing, China) and reverse-transcribed into cDNA using the HiScript II 1st Strand cDNA Synthesis Kit (+ gDNA wiper) (Vazyme). Real-time fluorescence quantitative PCR (RT-qPCR) was performed using CFX384 Real-Time PCR Detection System (Bio-Rad, Hercules, CA, USA) in conjunction with ChamQ Universal SYBR^®^ qPCR Master Mix (Vazyme). The relative expression levels of *RsAHLs* in radish tissues, abiotic stress samples, and overexpression plants were measured. The primer sequences are listed in **Supplementary Table 4**. Three biological replicates were used for each experiment. The radish *PRII* (RNA polymerase-II transcription factor) gene was used as the internal control (Xu et al., 2012) and the results were calculated using the 2^−ΔΔCt^ method.

### Statistical analyses

International Business Machine-Statistical Package for Social Sciences (IBM-SPSS) and Microsoft excel software were used for statistical analyses. All data were based on three independent biological replicates and expressed as the mean ± sd. Student’s *t*-test was used to calculate significant differences.

## Results

### Identification and characterization of RsAHLs in radish

To identify *AHLs* in radish, 29 AtAHL and 26 OsAHL protein sequences were used as queries for BlastP and HMM searches against the radish genome. A total of 52 AHL proteins were identified and named *RsAHL1–RsAHL52* (**Supplementary Table 1**), and then their physicochemical properties were analyzed. The *RsAHLs* ranged from 195 to 852 amino acids in length, with a relative molecular weight (MV) spanning between 20.86 and 94.62 kDa. Their isoelectric point (pI) was between 4.81and 9.83, while their aliphatic index ranged from 49.59 to 88.53. The grand average hydropathy (GRAVY) values of all *RsAHLs* were less than zero, indicating that *RsAHL* are strongly hydrophilic. Subcellular localization prediction showed that most of the *RsAHL* genes were located in the nucleus, followed by the chloroplast (**Supplementary Table 1**).

### Phylogenetic analysis of the RsAHL family

**Figure 1:**
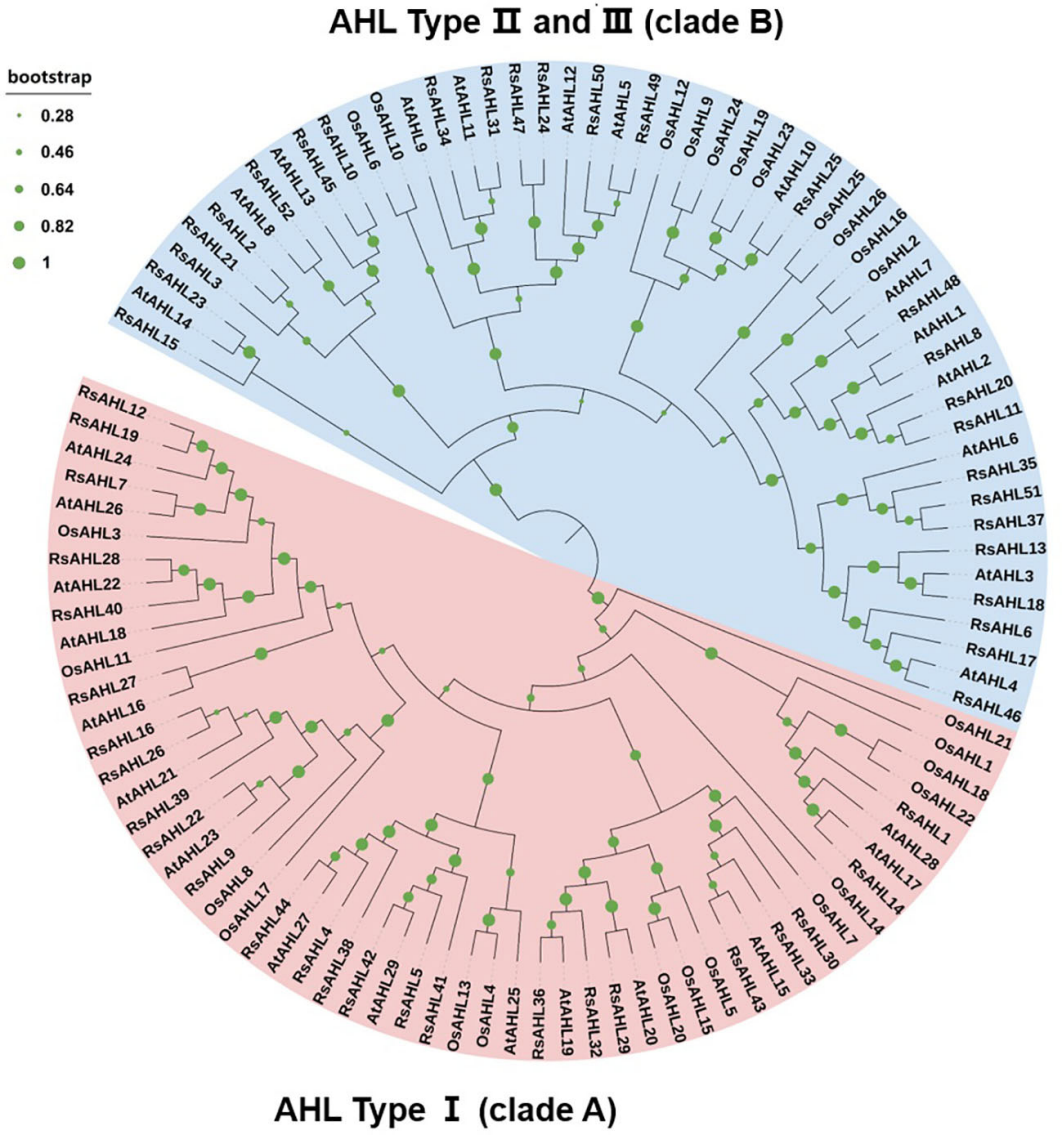
Phylogenetic tree of the *AT-hook motif nuclear localized* (*AHL*) family in radish, *Arabidopsis*, and rice. Different branch colors represent different subfamilies. The Rs- represents radish, the AT- represents *Arabidopsis*, and the LOC Os- represents rice.

To further infer the evolutionary relationships between RsAHL and other AHL proteins, we constructed phylogenetic trees using multiple sequence alignments of AHL proteins from radish (*Raphanus sativus*), *Arabidopsis*, and rice (*Oryza sativa* L.). The results showed that the *AHL* genes of these three species were divided into two clades: clades A and B. Within clade A, radish, *Arabidopsis*, and rice accounted for 25, 15, and 15 proteins, respectively, whereas within clade B, they accounted for 27, 14, and 11 proteins, respectively. Furthermore, the two independent clades A and B could be further divided into three types, with Type I belonging to clade A and Types II and III belonging to clade B (**Figure 1**). This result implies a high degree of phylogenetic consistency among *AHL* genes across different species and confirms the homology between the different species, suggesting that family members of the same branch may have similar functions.

### Chromosomal locations and collinearity of *RsAHLs*

**Figure 2:**
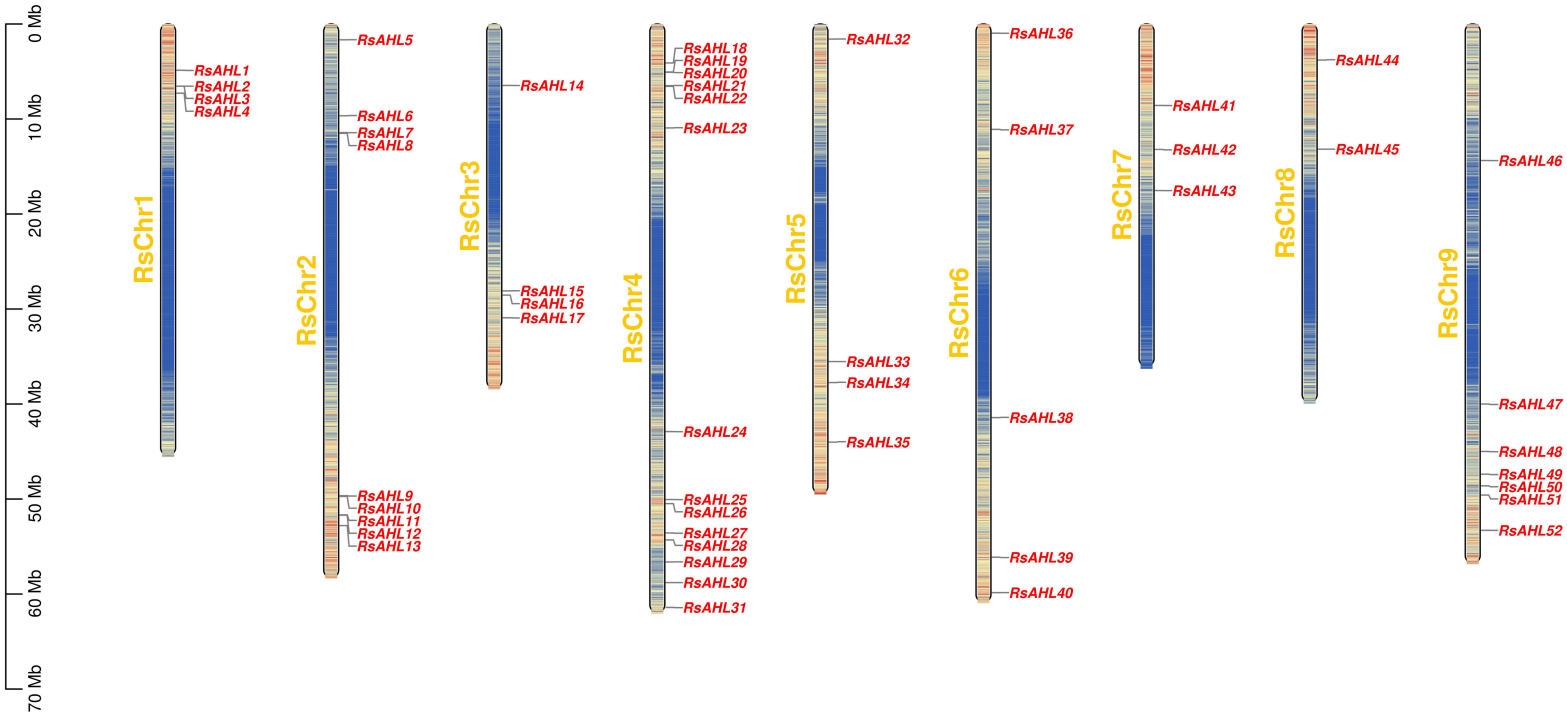
Arrangement of *Raphanus sativus AHLs* (*RsAHLs*) across nine chromosomes within radish. The name of each chromosome is on the left of the bar, while the gene name is on the right.

Based on the location information of each *RsAHL* gene, we arranged the positions of the 52 *RsAHL* genes across nine chromosomes of the radish genome, and chromosome mapping indicated that they were 52 *RsAHL* genes were unevenly distributed across the chromosomes (**Figure 2**). *RsAHLs* were mainly located on RsChr2, RsChr4, and RsChr9, which contained 9, 14, and 7 genes, respectively. Notably, only two *RsAHL* genes were found on RsChr8, namely *RsAHL44* and *RsAHL45*.

**Figure 3:**
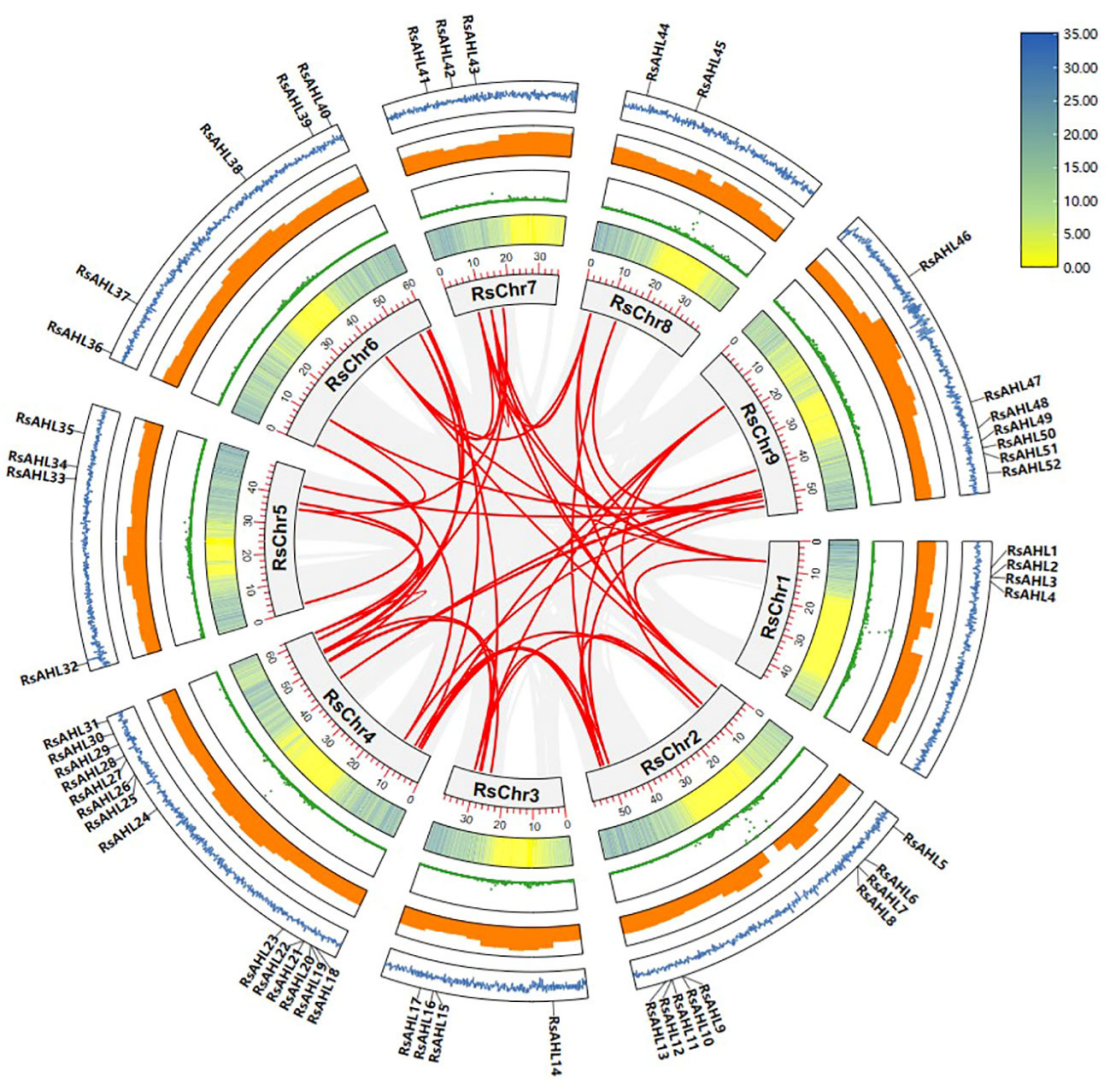
Collinearity analysis of the *RsAHL* gene family. The gray lines indicate all synteny blocks between each chromosome, and the red lines in the circle indicated duplicated *AHL* pairs. The circles from inside to outside represent gene density, N-ratio, GC-ratio and GC-skew.

Gene duplication was the most important cause of gene family expansion during plant evolution. To further study the evolutionary relationship of *RsAHL* in radish, gene duplication events were analyzed. And 49 duplications events were identified in 41 *RsAHL* genes. Among them, *RsAHL4* had the most collinearity relationship with other *RsAHLs*, including *RsAHL5*, *RsAHL38*, *RsAHL41*, *RsAHL42* and *RsAHL44*. However, 10 *RsAHLs* (*RsAHL1*, *RsAHL2*, *RsAHL3*, *RsAHL14*, *RsAHL15*, *RsAHL23*, *RsAHL25*, *RsAHL27*, *RsAHL29* and *RsAHL48*) had no collinearity relationship with the other *RsAHLs* (**Figure 3**; **Supplementary Table 2**). These results suggest that segment repeats play a crucial role in the expansion of the *RsAHL* family.

### Gene structure and conserved motif prediction analysis of *RsAHL* genes in radish

**Figure 4:**
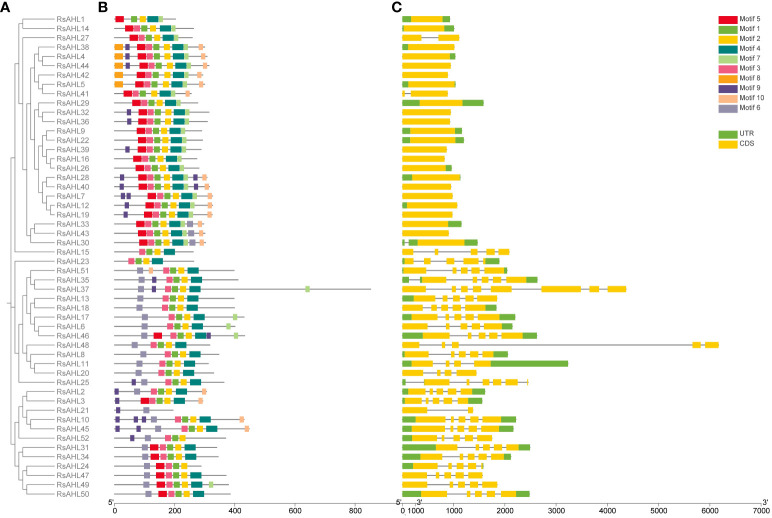
Phylogenetic tree, gene structure, and conserved motifs of RsAHLs in radish. **(A)** Phylogenetic tree was constructed using MEGA 10 in radish. **(B)** Motif structure diagram of RsAHL proteins. Different colors indicate different motif structures. **(C)** Gene structure diagram. The yellow box represents the coding sequence (CDS) region, the black thin line represents the intron, and the green represents the untranslated region (UTR).

To infer the evolutionary relationship between *RsAHL* genes, MEGA 10 was used to construct a phylogenetic tree of the 52 RsAHL protein sequences in radish (**Figure 4A**). We predicted the conserved protein motifs using the Multiple Expectation maximizations for Motif Elicitation (MEME) website (**Figure 4B**). We identified a total of 10 conserved motifs among the RsAHL proteins (**Supplementary Figure 1**; **Table 1**). These motifs ranged in length from 15 to 38 amino acids, with sites ranging from 5 to 51. Motifs 5 and 6 contained a conserved Arg-Gly-Arg core and belonged to the AT-hook motif family. In addition, motifs 5 and 6 were identified as Type I and Type II AT-hook motifs, respectively. Notably, we also identified the PPC domain (motif 2), which contains conserved Gly-Arg-Phe-Glu-Ile-Leu residues (Zhao et al., 2014). This motif has also been identified in maize, but not in soybean (Bishop et al., 2020; Wang et al., 2021b). Interestingly, almost all of the *RsAHL* genes contained motifs 1, 2, and 3, suggesting the consistency of the RsAHL protein sequences. Overall, the gene structure and motif prediction results indicated the consistency and evolutionary diversity of *AHL* genes in terrestrial plants.

**Table 1:** The detail information of RsAHL family conserved protein motifs.

Name	Sequence	E-value	Sites	Width
motif1	RQRGICVLSGTGTVSNVTLRQ	6.3e-573	51	21
motif2	VVTLEGRFEILSLSGSFLPPP	3.0e-557	51	21
moti3	LTPHVJEVNAGEDVVEKVMTF	6.5e-501	51	21
motif4	LSISLAGPQGQVVGGGVVGPLIAAGPVQVMAASFSNAA	2.6e-840	44	38
motif5	SVGRRPRGRPPGSKNKPKPPVIVTRDSPN	3.1e-447	30	29
motif6	KKKRGRPRKYAPDGSLALTLS	5.7e-203	25	21
motif7	YERLPLEEEEZZEGG	2.0e-152	30	15
motif8	MEGGYEQGGGASRYFHNLFRPEIHHQQQQ	4.5e-052	5	29
motif9	FKLHHHQQQQQQHNQ	6.0e-046	19	15
motif10	QQDPHLLYWGAGRPS	4.7e-034	19	15

Subsequently, we further analyzed the distribution of introns and exons in the *RsAHL* genes to explore the gene structure in radish (**Figure 4C**). The length of the *RsAHL* genes was between 824 and 6172 bp, among which the Type I genes were generally shorter than the Type II and III genes (**Supplementary Table 1**). Moreover, the numbers of introns and exons were diverse. We found that the genetic structure was similar for each type of gene. For example, Type I genes contained only one or two exons, and Types II and III contained more exons and introns than Type I, suggesting a diverse genetic structure of this subgroup. Therefore, we believe that Types II and III evolved from Type I. This result is consistent with that of the *AHL* gene family reported in maize (Bishop et al., 2020).

### Analysis of promoter cis-elements of the *RsAHL* family in radish

**Figure 5:**
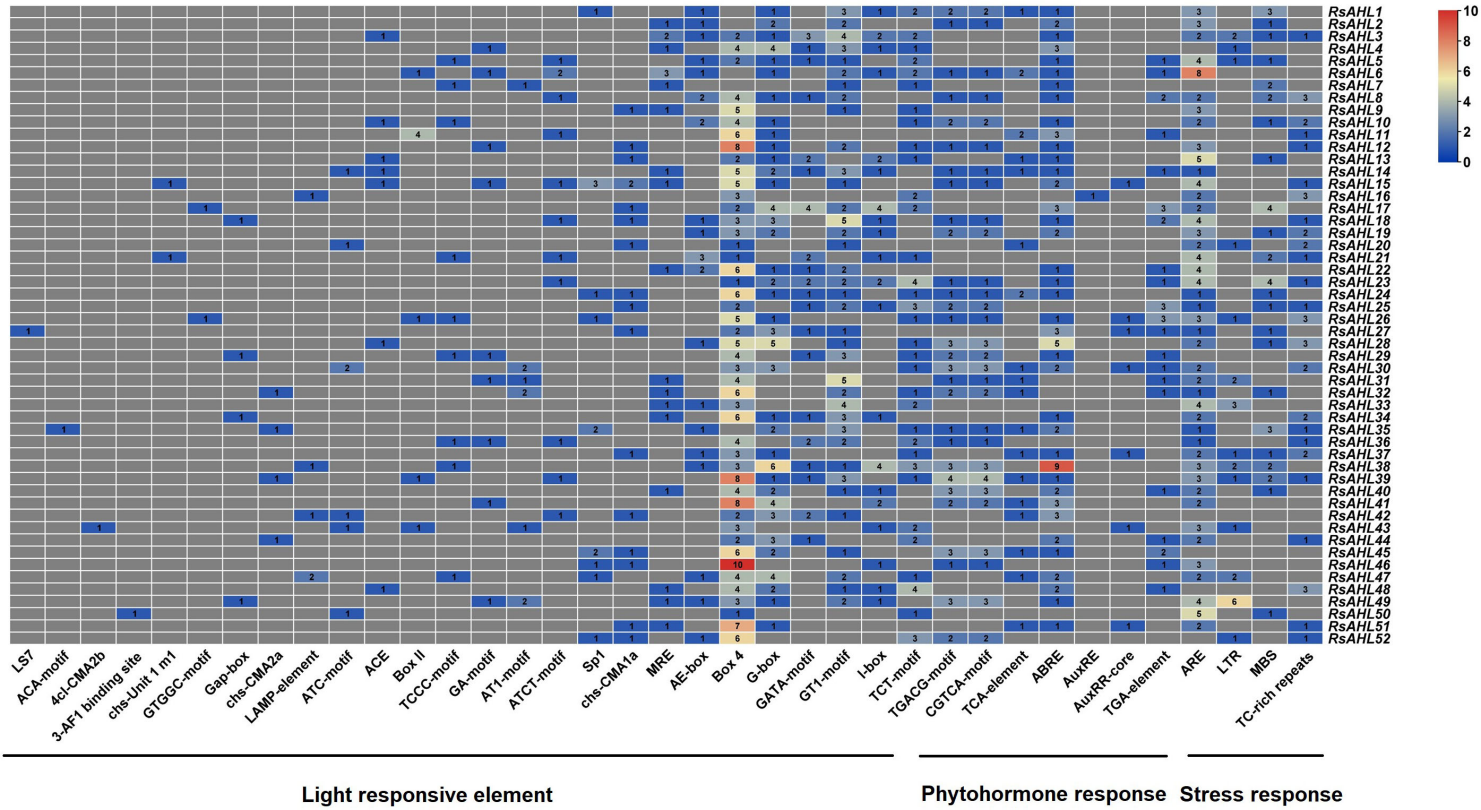
Number of cis-elements on the promoter of *RsAHL* genes in radish. Different colors represent the number of different cis-acting elements.

Cis-elements in promoters can be bound by transcription factors to regulate plant growth, development, defense, and stress responses by regulating gene expression. To infer the function of the *RsAHL* gene family, we used the PlantCARE database to perform cis-element analysis with a 2 kb promoter region of *RsAHL*. The functions of these cis-elements have also been identified (**Supplementary Table 3**). As shown in **Figure 5**, we found that all *RsAHL* promoters contained light response elements, including Box4, GT1-motif, G-box, TCT-motif, GATA-motif, I-box, AE-box, MRE, chs-CMA1a/2a, Sp1, ATCT-motif, AT1-motif, GA-motif, TCCC-motif, Box II, ACE, ATC-motif, LAMP-element, Gap-box, GTGGC-motif, chs-Unit 1, 3-AF1 binding site, 4 cl-CMA2b, ACA-motif, and LS7. The Box4 cis element exists in almost all *RsAHL* genes, with *RsAHL46* containing the highest number. This suggests that *RsAHL* genes are likely regulated by light. Approximately 86.5% of *RsAHL* family members contained anaerobic induction elements (ARE), with *RsAHL6* containing the highest number. Seven cis-elements were associated with hormonal responses: abscisic acid (ABRE and TCA-element), methyl jasmonate or MeJA (CGTCA-motif, TGACG-motif), and auxin (AuxRR-core, TGA-element, and AuxRE). Additionally, we found that 26.9%, 44.2%, and 48.1% of *RsAHL* family members contained low-temperature elements (LTR), drought induction elements (MBS), and defense and stress responsiveness (TC-rich repeats), respectively. These results indicate that the *RsAHL* gene family plays an important role in regulating plant growth, hormone responses, and abiotic stress responses.

### Tissue specific expression of the *RsAHL* family in radish

**Figure 6:**
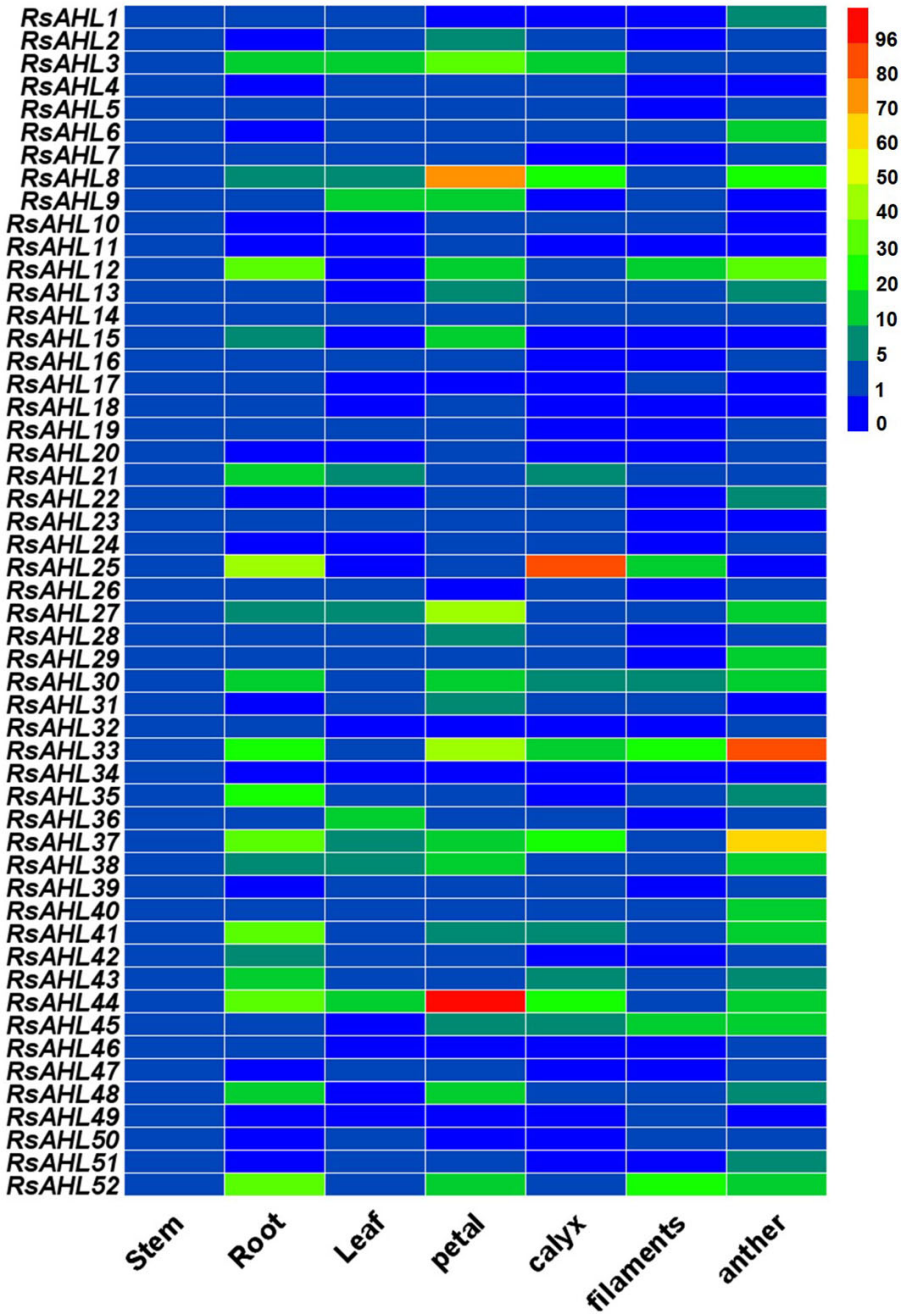
Relative expression level of the *RsAHL* family in various tissues of radish. Different colored boxes indicate different levels of expression.

To study the expression of *RsAHLs* in various radish tissues, we extracted RNA from the taproots, stems, leaves, petals, calyx, filaments, and anthers of radish and detected the relative expression levels of *RsAHLs* among them (**Figure 6**). The results showed that *RsAHLs* had different expression patterns in different tissues of the radish plant. The relatively highest expression of *RsAHL* genes was observed in the anthers, petals, and taproots. In particular, *RsAHL44* expression was the highest in petals, *RsAHL33* expression was the highest in anthers, and *RsAHL25* expression was the highest in taproots and calyxes. On the contrary, *RsAHL11*, *RsAHL17*, *RsAHL18*, *RsAHL20*, *RsAHL32*, *RsAHL34*, *RsAHL46* and *RsAHL49* exhibited relatively low expression across various organs. In addition, the expression levels of the *RsAHL* family in filaments were lower than those in other flower organs. These results indicate that *RsAHL* family is mainly expressed in the taproots and floral organs, suggesting the potential function of *RsAHL* genes in taproots and flowers.

### Expression pattern of *RsAHL* family under hormone stress

**Figure 7:**
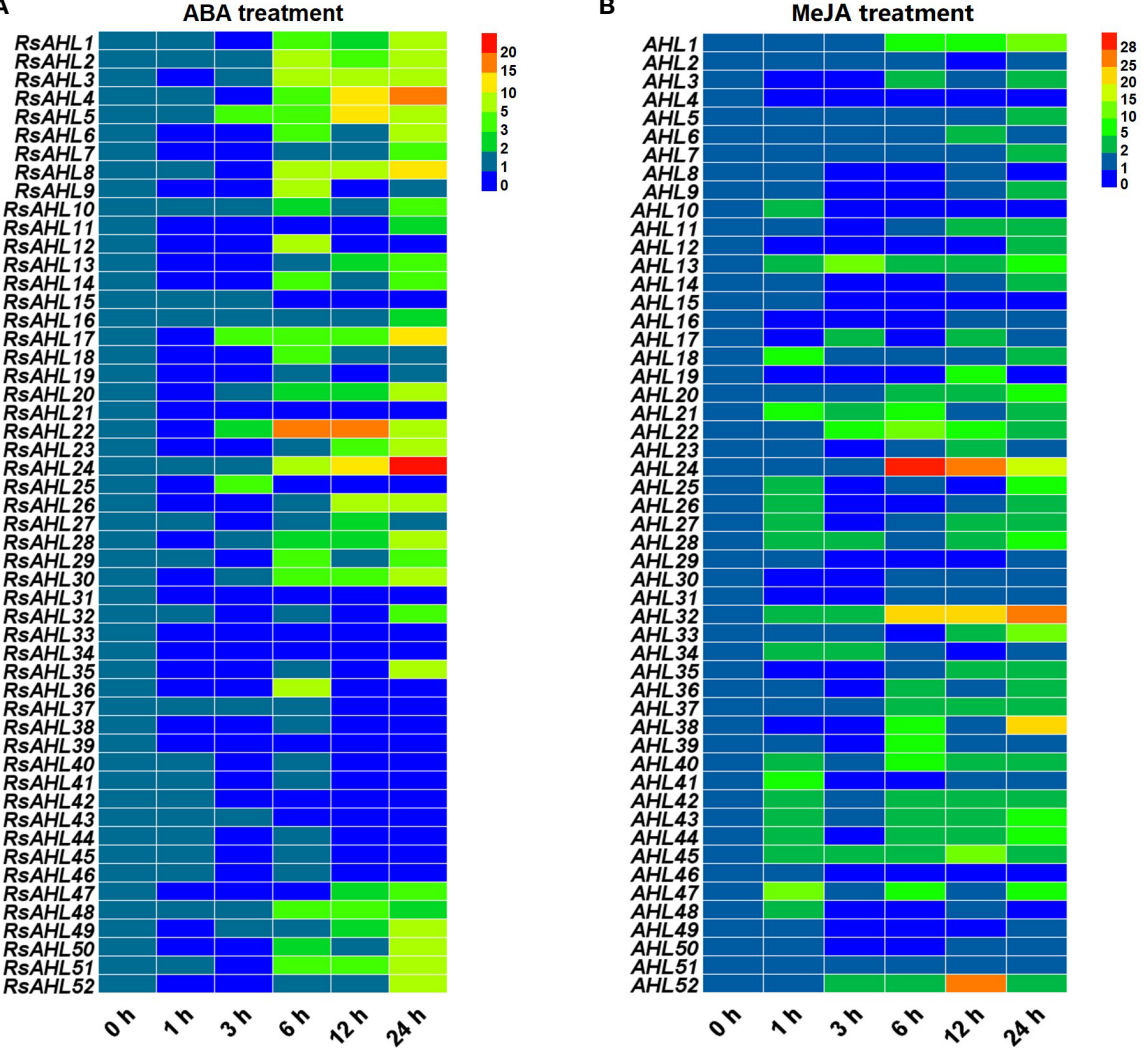
Relative expression level of the *RsAHL* family under hormone treatment. **(A)** Relative expression level of *RsAHLs* in radish seedlings after abscisic acid (ABA) treatment. **(B)** Relative expression level of *RsAHLs* in radish seedlings after methyl jasmonate (MeJA) treatment. Here, 0 h, 1 h, 3 h, 6 h, 12 h, and 24 h represent the time points of sampling after treatment. Different colored boxes indicate different levels of expression.

To further explore the expression pattern of the *RsAHL* family in radish under hormonal stress, qRT-PCR was performed to analyze the 52 *RsAHL* genes under different stress treatments. Based on the analysis of cis-acting elements in the promoter, MeJA and ABA were predicted to induce the strongest hormone responses. Subsequently, MeJA (200 μM) and ABA (10 μM) were further used to treat radish seedlings (**Figure 7**). These results showed that *RsAHL* genes presented varying responses to the MeJA and ABA treatments. Under ABA treatment, 14 *RsAHL* genes were significantly upregulated after 6 h, with *RsAHL4*, *RsAHL8*, and *RsAHL24* showing a gradual increase in expression. *RsAHL5*, *RsAHL17* and *RsAHL22* were upregulated at 3 h and reached their highest expression levels at 12, 24, and 6 h, respectively. In contrast, *RsAHL21*, *RsAHL31*, *RsAHL33* and *RsAHL39* were down-regulated after ABA treatment, whereas *RsAHL15* and *RsAHL43* were down-regulated after 6 h (**Figure 7A**). Under MeJA treatment, the expression of *RsAHL13*, *RsAHL21*, *RsAHL28*, *RsAHL32* and *RsAHL45* were continuously upregulated at 1 h. *RsAHL22* and *RsAHL52* were upregulated at 3 h, *RsAHL22* reached its highest level at 6 h and then decreased, while *RsAHL52* reached its highest value at 12 h and then decreased. *RsAHL24* expression was upregulated at 6 h, reached its highest level, and then decreased thereafter. In contrast, the expression of *RsAHL4* was downregulated immediately after treatment, whereas that of *RsAHL10*, *RsAHL15* and *RsAHL46* began to decline after 3 h. Notably, *RsAHL51* did not change significantly after treatment, indicating that it did not respond to MeJA induction (**Figure 7B**). Therefore, the expression of *RsAHLs* in most radish samples were significantly upregulated by the ABA and MeJA treatments. Particularly, the expression of *RsAHLs* was significantly upregulated 6 and 1 h after ABA and MeJA induction, respectively.

### Expression pattern of *RsAHL* family under abiotic stress conditions

**Figure 8:**
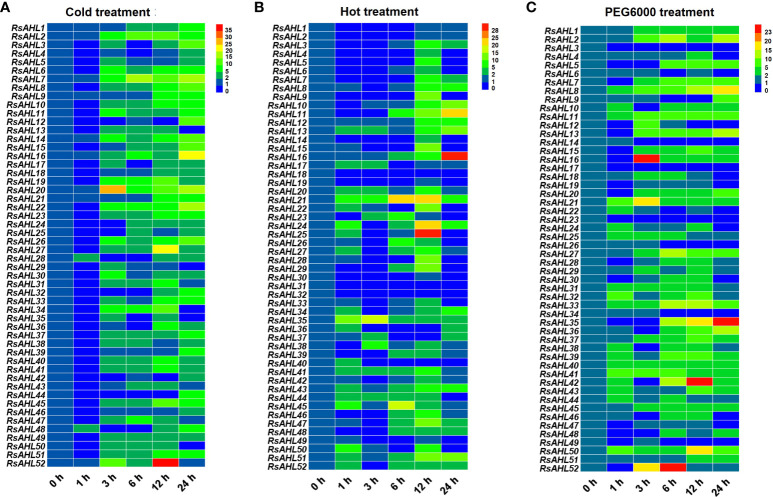
Expression patterns of *RsAHLs* under various abiotic stress. **(A)** Relative expression level of *RsAHLs* in radish seedlings after cold treatment (4°C). **(B)** Relative expression level of *RsAHLs* in radish seedlings after hot treatment (42°C). **(C)** Relative expression level of *RsAHLs* in radish seedlings after polyethylene glycol PEG6000 treatment. Here, 0 h, 1 h, 3 h, 6 h, 12 h, and 24 h represent the time points of sampling after treatment. Different colored boxes indicate different levels of expression.

To investigate the roles of the *RsAHL* family under abiotic stress, the expression levels of *RsAHLs* were determined after cold (4°C), hot (42°C) and polyethylene glycol PEG6000 (20%) treatment. Under the 4°C treatment, the expression of most *RsAHL* genes was significantly upregulated after 3 h, and reached the highest level at 24 h. The expression of *RsAHL20* reached its highest level at 3 h, while that of *RsAHL52* reached its highest level at 12 h (**Figure 8A**). At 42 °C treatment, most of the *RsAHL* genes were significantly upregulated and reached their highest expression at 12 h. The expression levels of *RsAHL11*, *RsAHL16*, and *RsAHL51* gradually increased after 6 h of treatment, whereas the expression levels of *RsAHL21* and *RsAHL35* increased after 1 h. In contrast, the expression levels of *RsAHL18*, *RsAHL31* and *RsAHL32* were downregulated after treatment (**Figure 8B**). Most *RsAHLs* were significantly upregulated after PEG6000 treatment. The expression levels of *RsAHL8*, *RsAHL11*, *RsAHL21*, *RsAHL33*, *RsAHL40*, *RsAHL41*, and *RsAHL50* were consistently upregulated after treatment. Notably, 10 *RsAHL* genes were consistently upregulated after 3 h of treatment and *RsAHL35* and *RsAHL36* were gradually upregulated after 6 h of treatment. However, *RsAHL3*, *RsAHL17*, and *RsAHL23* were downregulated after treatment (**Figure 8C**). These results suggest that *RsAHL* plays an important role in abiotic stress.

### Overexpression of *RsAHL14* inhibited the growth of tomato plants

**Figure 9:**
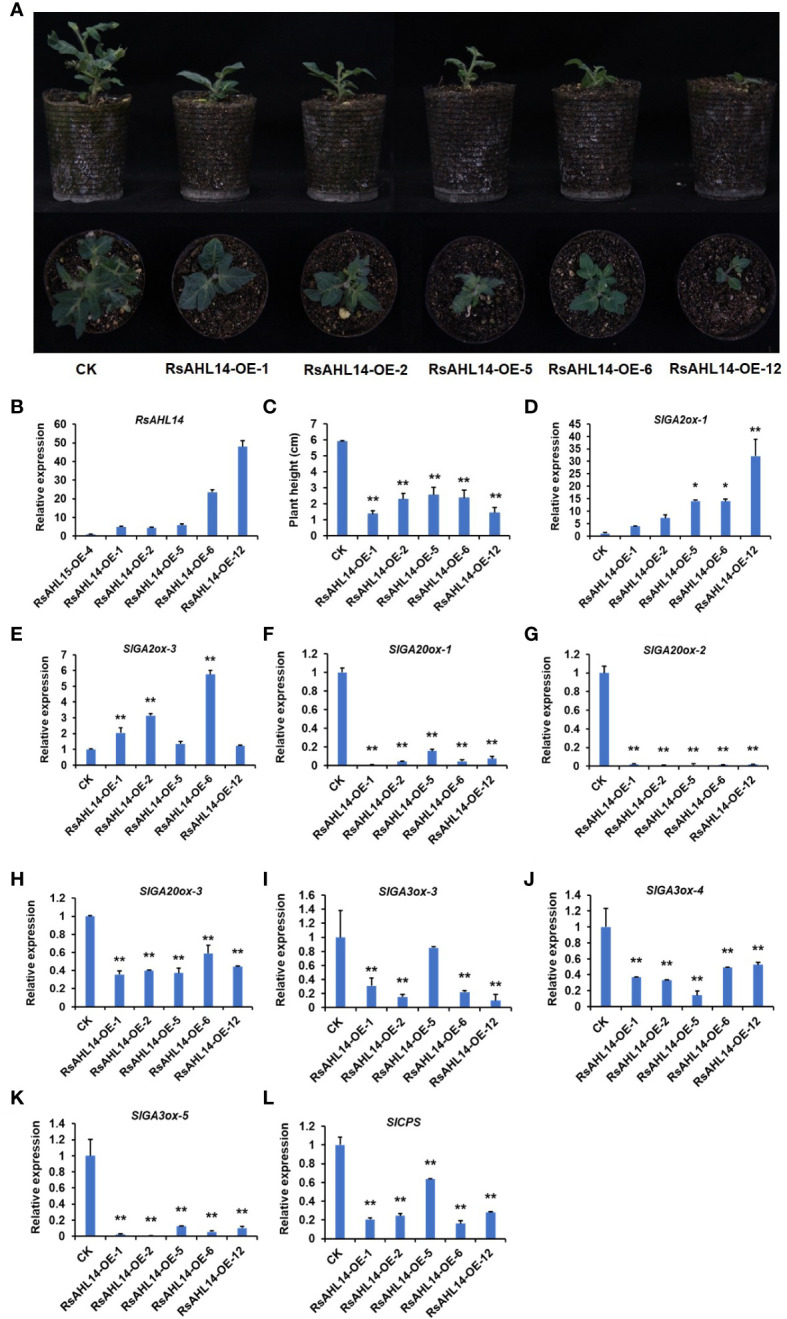
Overexpression of *RsAHL14* causes tomato dwarfing by regulating the expression of GA synthesis and degradation genes. **(A)** Phenotypes of the *RsAHL14*-overexpression lines and CK in tomato. **(B)** Relative expression level of *RsAHL14* in tomato leaves of the overexpression lines and CK. **(C)** Plant height of the *RsAHL14*-overexpression lines and CK. Relative expression level of *SlGA2ox-1* **(D)**, *SlGA2ox-3* **(E)**, *SlGA20ox-1* **(F)**, *SlGA20ox-2* **(G)**, *SlGA20ox-3* **(H)**, *SlGA3ox-3* **(I)**, *SlGA3ox-4* **(J)**, *SlGA3ox-5* **(K)** and *SlCPS* **(L)** in tomato leaves of the overexpression lines and CK. The asterisks indicate significant difference (**P ≤ 0.01, *P ≤ 0.05).

To identify the biological function of the *RsAHL* family, we constructed overexpression vectors of several *RsAHL* genes and used MicroTom as the background material for genetic transformation. Using vector forward and gene reverse primers to detect the positive of transgenic materials, we obtained RsAHL14-overexpression positive plants (**Supplementary Figure 2**), with the RsAHL14-OE-1/-2/-5/-6/-12 lines used for further experiments. In the observation of the phenotype of the positive transgenic tomato plants, it was found that plants with *RsAHL14* overexpression were dwarfed during the developmental process compared to the control (CK) (**Figure 9A**). Further, the plant height was significantly lower in *RsAHL14*-overexpression lines compared with CK (**Figure 9C**). To analyze the relative expression of *RsAHL14* in the overexpression lines, RsAHL14-OE-4 (which showed low expression of RsAHL14 in positive materials) was used as the control. The results showed that the relative expression levels of *RsAHL14* in the leaves of the RsAHL14-OE-1/-2/-5/-6/-12 lines were 5.0-, 4.4-, 5.9-, 23.6-, and 48.0-fold higher than that in the level of RsAHL14-OE-4, respectively (**Figure 9B**). Notably, previous studies have shown that changes in the gibberellin (GA) content in plants can cause dwarfing.

To determine whether changes in GA lead to dwarfing, the expression of genes involved in GA biosynthesis and degradation pathways was detected in *RsAHL14* overexpression plants. Among these, ent-Copalyl diphosphatase (CPS), ent-Kaurene synthase (KS), ent-Kaurene oxidase (KO), GA20-oxidase (GA20ox), and GA3-oxidase (GA3ox) are key enzymes in the GA biosynthetic pathway, while GA2-oxidase (GA2ox) is a key enzyme in the degradation pathway. As expected, compared with the CK, the relative expression levels of *GA2ox-1* and *GA2ox-3* were significantly increased in the RsAHL14-OE plants (**Figures 9D, E**), while those of *GA20ox-1*, *GA20ox-2*, *GA20ox-3*, *GA3ox-3*, *GA3ox-4*, *GA3ox-5* and *CPS* were significantly decreased (**Figures 9F-L**). However, the expression levels of other GA-related genes were irregular in the overexpression lines (**Supplementary Figure 3**). This suggests that GA synthesis was hindered in *RsAHL14*-overexpression lines, the degradation rate was accelerated, and the resulting plants were dwarfed.

## Discussion

Radish is an important root crop and one of the main autumn and winter vegetables. *AHLs* play an important role in plant growth, development, and stress resistance in *Arabidopsis*, maize, rice, cotton, and soybeans (Kim et al., 2011; Zhao et al., 2014; Bishop et al., 2020; Zhao et al., 2020; Wang et al., 2021b). However, the identification of AHL proteins in radish has not yet been reported. In this study, we performed a genome-wide analysis of *AHLs* in radish and identified 52 *RsAHLs*. The phylogenetic analysis revealed that *RsAHLs* could be divided into two clades (A and B) and three types (I, II, and III) (**Figure 1**), which is consistent with the results obtained for other land plants. Notably, we identified a PPC domain (motif 2) in RsAHL proteins (**Figure 4**; **Supplementary Figure 1**; **Table 1**); however, no such domain was found in soybeans (Wang et al., 2021b). The PPC domains could interact with each other or with other transcription factors to regulate transcriptional activation (Zhao et al., 2013; Seo and Lee, 2021), suggesting a diverse biological function for *RsAHLs*.

In soybean and maize, AHL proteins are present in multiple organelles, including the nucleus, cytoplasm, and chloroplasts (Bishop et al., 2020; Wang et al., 2021b). Similar results were obtained for radish in this study (**Supplementary Table 1**), suggesting that the subcellular localization of *AHLs* is conserved across various species. All *RsAHL* gene families were unevenly distributed across nine chromosomes (**Figure 2**). Collinearity analysis showed that multiple gene duplication events occurred in the *RsAHL* genes within the radish genome (**Figure 3**; **Supplementary Table 2**), suggesting that *RsAHLs* expanded through gene duplication and gained and lost their functions.

Several studies have demonstrated the involvement of *AHL* genes in various stress responses (Yadeta et al., 2011; Zhou et al., 2016; Wong et al., 2019). Cis-elements in promoters are believed to influence plant growth, development, and stress responses (Yamaguchi-Shinozaki and Shinozaki, 2006; Himmelbach et al., 2010). Analysis of the cis element of the *RsAHL* promoter is conducive to a comprehensive understanding of its potential function. Most *RsAHLs* contained anaerobic induction elements and responded to ABA, MeJA, and auxins (**Figure 5**). In an anaerobic environment, plant root development is blocked, and epidermal cells are damaged or killed, leading to pathogen infection (Kuan and Erwin, 1980). Notably, all *RsAHL* genes can be photoinduced because their promoters contain light-responsive elements. In addition, some *RsAHLs* respond to low temperature and drought stress. In the study of grape and soybean, all grape and soybean *AHL* gene promoters contained light response elements, hormone response elements, and stress response elements (Li et al., 2021; Wang et al., 2021b). This suggests that the *AHL* genes in radish and other plant species affect plant growth, development, and stress response.

The *AHL* family is widely distributed in plants and is vital in the regulation of flower, hypocotyl, root, and leaf development (Street et al., 2008; Xiao et al., 2009; Yun et al., 2012; Tayengwa et al., 2020; Seo and Lee, 2021; Zhou et al., 2022). To better understand the specific expression patterns of *RsAHL* in radish, we analyzed the relative expression levels of 52 *RsAHLs* in different tissues. We found that *RsAHLs* were highly expressed in flower organs and taproots compared to that in other tissues (**Figure 6**). *AHL3*, *AHL4*, *AHL18*, and *OsAHL1* regulate the development of root (Zhou et al., 2013, 2016; Sirl et al., 2020). Whereas, *DP1*, *AHL16/TEK*, *AHL20*, *AHL21/GIK*, *AHL22*, *AHL23*, *AHL27*, *Baf1* regulates the development of various flower organs (Ng et al., 2009; Xiao et al., 2009; Gallavotti et al., 2011; Jin et al., 2011; Yun et al., 2012; Jia et al., 2015; Tayengwa et al., 2020). These results indicate that *RsAHLs* play a crucial role in the development of flowers and taproots in radish plants. In particular, *DcAHLc1* plays an important role in the development of storage root in carrot (Macko-Podgórni et al., 2017).

Under biotic and abiotic stresses, the transcription level of *AHL* is significantly affected. For example, the overexpression of *OsAHL1* can improve rice drought tolerance and resistance, participate in the oxidative stress response, and regulate leaf chlorophyll content (Zhou et al., 2016). Similarly, under the PEG treatment, the expression of grape *AHL* genes was induced by different degrees of stress (Li et al., 2021). We further found that radish *RsAHL* genes were also induced to different degrees under PEG treatment. Similar results were found after the 42°C heat treatment and 4°C cold treatment (**Figure 8**). A similar study induced change in *PtrAHL12* and *PtrAHL17* expression through ABA and drought treatments, respectively (Wang et al., 2021a). Our results showed that *RsAHL* gene expression was significantly upregulated after 6 h of ABA treatment, and after 6 and 12 h of MeJA treatment (**Figure 7**). These results imply a multifaceted role of *RsAHL* genes in plant stress responses and reveal that further examination of the biological functions of these genes is warranted to help improve the adaptability of radish under various abiotic stress conditions.

**Figure 10:**
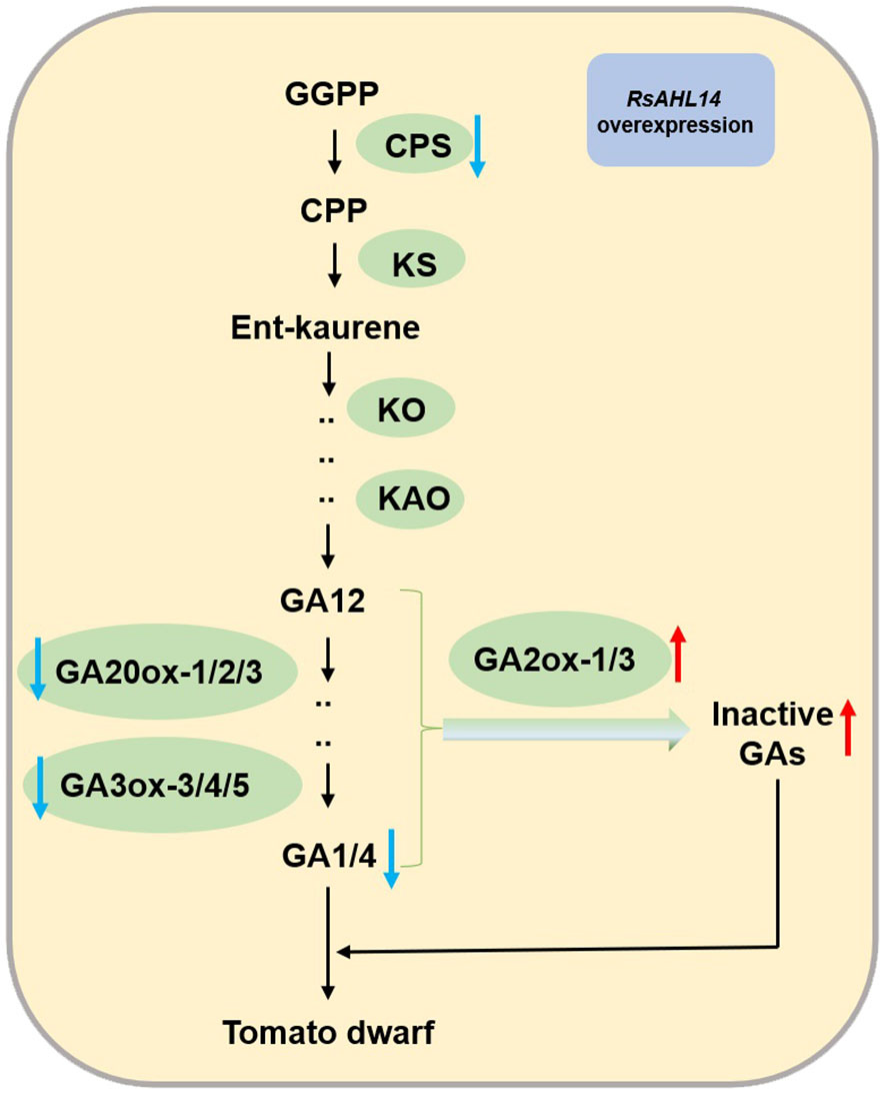
Model for the effects of *RsAHL14* heterologous overexpression on the regulation of GA and dwarf phenotypes in tomato. GGPP, geranylgeranylpyrophosphate; CPP, copalyl pyrophosphate. Blue arrows represent a decrease, and red arrows represent an increase.

Furthermore, we found that *RsAHL14*-overexpression in tomato caused plant dwarfing, and numerous studies have shown that changes in the plant GA content can also cause dwarfing. For example, the dwarfing phenotypes of *Arabidopsis ga3*, maize *dwarf3*, and rice *dwarf35* mutants are due to the transcription of GA synthetase P450 monooxygenase (P450) (Winkler and Helentjaris, 1995; Helliwell et al., 1998; Itoh et al., 2004). The GA content in plants is determined by synthesis and degradation pathways. In the present study, the relative expression levels of seven GA biosynthetic genes were significantly decreased, whereas the expression levels of *SlGA2ox-1/-3* in the degradation pathway were significantly increased in *RsAHL14*-overexpression tomato plants (**Figure 9**). AGF1 (an AT-hook protein) regulates the GA balance by negatively regulating *AtGA3ox-1* (Matsushita et al., 2007), suggesting that *AHL* genes play an important role in maintaining GA homeostasis. According to Yamaguchi’s study (Yamaguchi, 2008), a simple model of *RsAHL14* expression in tomato plants was developed, and it was shown to cause changes in the expression of GA synthesis and degradation genes, which may lead to an imbalance of active and inactive GA content and ultimately lead to a tomato dwarf phenotype (**Figure 10**). However, the specific regulatory mechanism of *RsAHL14* on GA needs to be further studied.

## Conclusions

In this study, a total of 52 *RsAHL* genes were identified in radish, and they unevenly distributed on 9 chromosomes. The phylogenetic tree divided these genes into two clades and three types based on the AT-hook motif and PPC domain. Furthermore, the cis-acting elements of 2 kb promoter regions of *RsAHL* genes and their expression in different tissue were investigated, and their response to abiotic stress and hormones was also determined. Meanwhile, heterologous expression of *RsAHL14* can induce dwarfing in tomato plants by regulating the expression of GA-related genes, thus suggesting the important relationship between *RsAHL* genes and plant hormones, especially GA. These results will provide a basis for further exploring the biological functions of the *RsAHL* family in growth regulation and stress responses.

## Data availability statement

The original contributions presented in the study are included in the article/**Supplementary Material**. Further inquiries can be directed to the corresponding author/s.

## Author contributions

WC: Conceptualization, Data curation, Funding acquisition, Software, Visualization, Writing – original draft. LCh: Data curation, Investigation, Writing – review & editing. LCu: Resources, Supervision, Writing – review & editing. ZL: Investigation, Software, Validation, Writing – original draft. WY: Conceptualization, Funding acquisition, Supervision, Writing – review & editing.
