# Supplementary material for: Genome-wide analysis of radish AHL gene family and functional verification of RsAHL14 in tomato
Source: Front Plant Sci. 2024 May 30;15:1401414. doi: 10.3389/fpls.2024.1401414 (PMC11169806; doi:10.3389/fpls.2024.1401414)
Supplement: Supplementary file 1 [file DataSheet_1.docx]

Supplementary Material

Genome-wide analysis of the radish *AHL* gene family and functional verification of *RsAHL14* in tomato

Weifang Chen, Leifu Chen, Lei Cui, Zhixiong Liu and Weiling Yuan ^*^

*** Correspondence:**

Weiling Yuan

ywiing2021@hbaas.com

Supplementary Figure 1 to 3

Supplementary Table 1 to 4


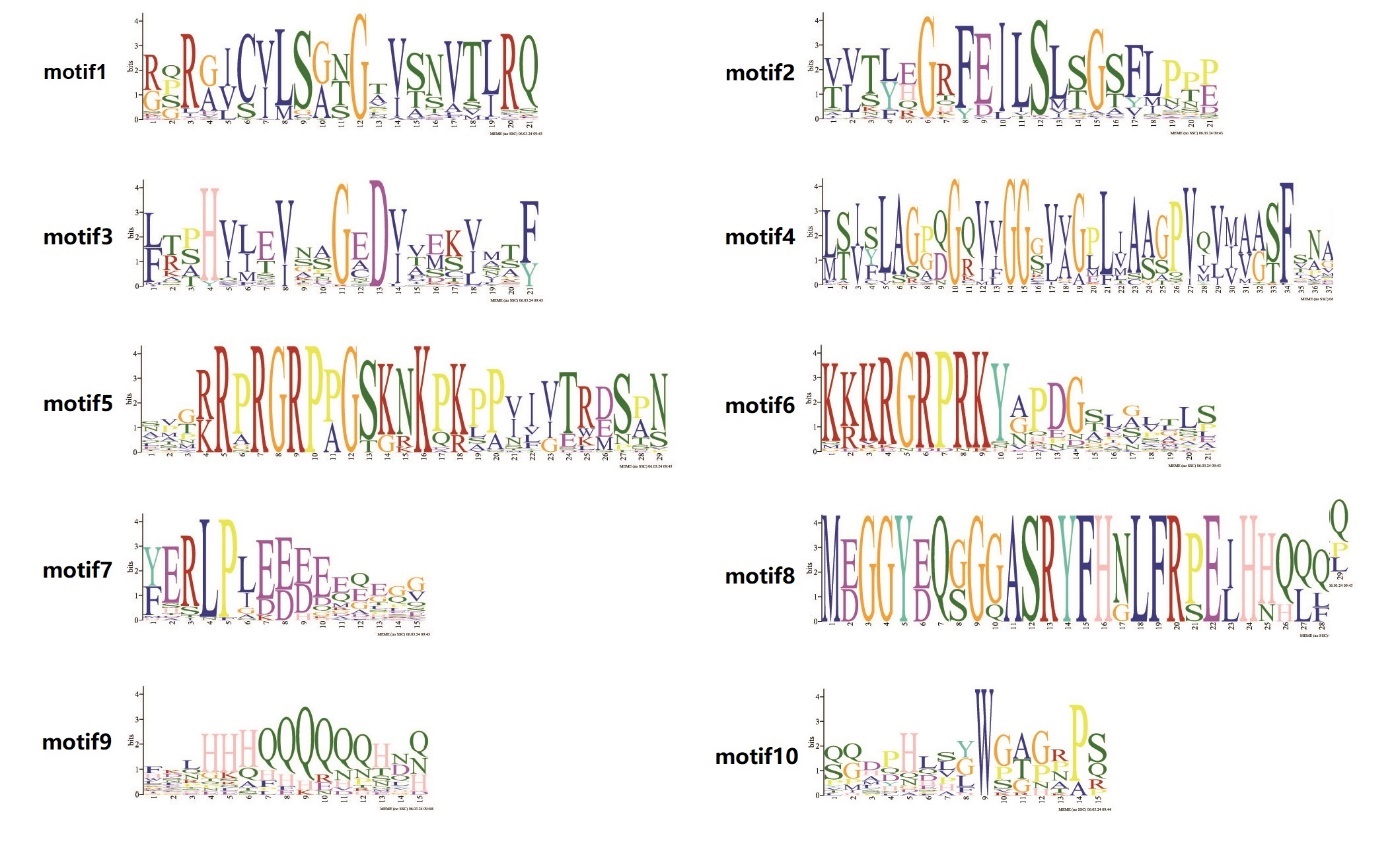


**Supplementary Figure 1.** Conserved motif of RsAHL protein sequence.


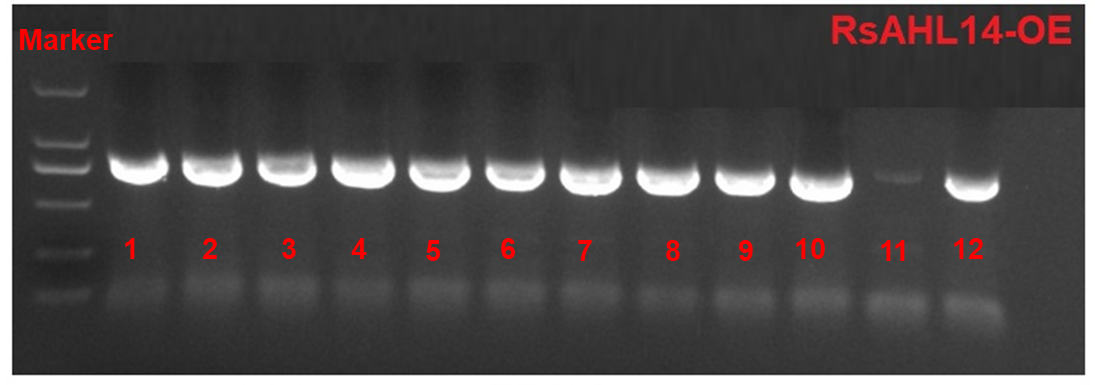


**Supplementary Figure 2.** The positive detection of RsAHL14-OE transgenic material was amplified by PCR. gDNA was extracted from young leaves and used as amplification template. Vector forward primers (35S) and gene direction primers were used for amplification. Marker strips from top to bottom are: 2000, 1500, 750, 500, 200 and 100 bp, respectively.


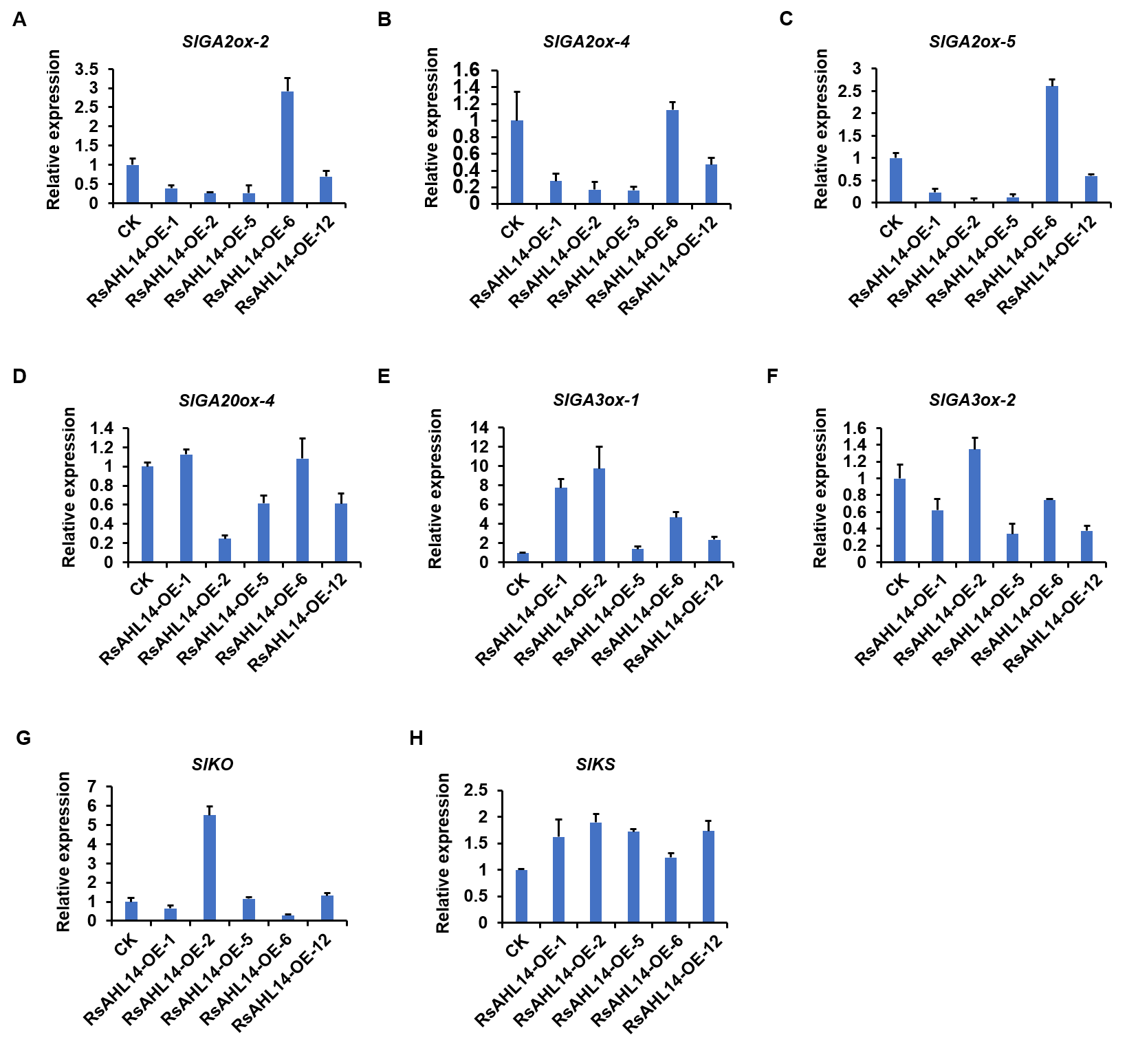


**Supplementary Figure 3.** Relative expression level of *SlGA2ox-2* (**A**), *SlGA2ox-4* (**B**), *SlGA2ox-5* (**C**), *SlGA20ox-4* (**D**), *SlGA3ox-1* (**E**), *SlGA3ox-2* (**F**), *SlKO* (**G**) and *SlKS* (**H**) in overexpression lines and CK.

**Supplementary Table 1.** The detail information of *AT-hook motif* gene family.

| Gene Name | Gene ID | Chromosome position | CDS length (bp) | Protein length (aa) | Molecular weight (kDa) | Isoelectric point (PI) | Aliphatic index | GRAVY | Subcellular location |
| --- | --- | --- | --- | --- | --- | --- | --- | --- | --- |
| *RsAHL1* | GWHPCBIT000972 | Chr1:4869731-4870660 | 612 | 203 | 20.93 | 6.83 | 78.28 | -0.171 | chlo |
| *RsAHL2* | GWHPCBIT001294 | Chr1:6546707-6548321 | 921 | 306 | 32.00 | 6.32 | 70.16 | -0.502 | nucl |
| *RsAHL3* | GWHPCBIT001295 | Chr1:6549166-6550724 | 885 | 294 | 30.48 | 8.58 | 62.72 | -0.533 | nucl |
| *RsAHL4* | GWHPCBIT001424 | Chr1:7283607-7284640 | 927 | 308 | 31.43 | 6.84 | 63.28 | -0.567 | nucl |
| *RsAHL5* | GWHPCBIT003264 | Chr2:1664381-1665418 | 903 | 300 | 30.52 | 7.11 | 67.57 | -0.498 | nucl |
| *RsAHL6* | GWHPCBIT004007 | Chr2:9659875-9662023 | 1206 | 401 | 42.85 | 5.37 | 59.6 | -0.585 | golg |
| *RsAHL7* | GWHPCBIT004145 | Chr2:11427927-11428907 | 981 | 326 | 35.08 | 6.83 | 58.34 | -0.7 | nucl |
| *RsAHL8* | GWHPCBIT004149 | Chr2:11508132-11510191 | 1044 | 347 | 36.02 | 9.23 | 71.93 | -0.254 | plas |
| *RsAHL9* | GWHPCBIT006398 | Chr2:49647280-49648442 | 873 | 290 | 29.30 | 5.8 | 76.28 | -0.185 | nucl |
| *RsAHL10* | GWHPCBIT006409 | Chr2:49725812-49728031 | 1296 | 431 | 45.75 | 9.46 | 51.14 | -0.844 | nucl |
| *RsAHL11* | GWHPCBIT006745 | Chr2:51663698-51666932 | 939 | 312 | 32.45 | 9.39 | 65 | -0.372 | plas |
| *RsAHL12* | GWHPCBIT006748 | Chr2:51702500-51703571 | 981 | 326 | 34.57 | 6.25 | 56.81 | -0.671 | chlo |
| *RsAHL13* | GWHPCBIT006962 | Chr2:52794794-52796642 | 1197 | 398 | 42.46 | 4.81 | 55.68 | -0.627 | nucl |
| *RsAHL14* | GWHPCBIT008531 | Chr3:6468382-6469391 | 792 | 263 | 27.77 | 8.37 | 69.28 | -0.293 | cyto |
| *RsAHL15* | GWHPCBIT009379 | Chr3:28091518-28093605 | 789 | 262 | 27.82 | 7.81 | 81.79 | -0.181 | nucl |
| *RsAHL16* | GWHPCBIT009444 | Chr3:28535941-28536765 | 825 | 274 | 28.18 | 6.35 | 70.8 | -0.225 | nucl |
| *RsAHL17* | GWHPCBIT009783 | Chr3:30917307-30919510 | 1296 | 431 | 45.42 | 5.41 | 60.63 | -0.548 | chlo |
| *RsAHL18* | GWHPCBIT011894 | Chr4:4100912-4102748 | 1200 | 399 | 42.11 | 5.41 | 56.57 | -0.534 | nucl |
| *RsAHL19* | GWHPCBIT012044 | Chr4:5052493-5053470 | 978 | 325 | 34.17 | 6.1 | 58.49 | -0.663 | chlo |
| *RsAHL20* | GWHPCBIT012047 | Chr4:5084508-5085953 | 993 | 330 | 34.52 | 9.32 | 64.7 | -0.36 | plas |
| *RsAHL21* | GWHPCBIT012243 | Chr4:6486565-6487944 | 588 | 195 | 20.86 | 9.64 | 49.59 | -1.079 | nucl |
| *RsAHL22* | GWHPCBIT012254 | Chr4:6562248-6563448 | 882 | 293 | 29.55 | 6.18 | 74.81 | -0.209 | nucl |
| *RsAHL23* | GWHPCBIT012947 | Chr4:10937244-10939136 | 795 | 264 | 27.87 | 6.24 | 74.62 | -0.097 | nucl |
| *RsAHL24* | GWHPCBIT014574 | Chr4:42900137-42901718 | 867 | 288 | 31.02 | 9.71 | 58.3 | -0.535 | nucl |
| *RsAHL25* | GWHPCBIT015444 | Chr4:50049099-50051554 | 1095 | 364 | 37.86 | 9.83 | 60.27 | -0.563 | nucl |
| *RsAHL26* | GWHPCBIT015528 | Chr4:50483185-50484147 | 846 | 281 | 28.75 | 6.24 | 69.07 | -0.27 | nucl |
| *RsAHL27* | GWHPCBIT016012 | Chr4:53569450-53570559 | 780 | 259 | 27.73 | 9.55 | 88.53 | -0.189 | chlo |
| *RsAHL28* | GWHPCBIT016151 | Chr4:54304399-54305531 | 927 | 308 | 32.68 | 6.52 | 68.73 | -0.572 | nucl |
| *RsAHL29* | GWHPCBIT016411 | Chr4:56621264-56622848 | 834 | 277 | 28.79 | 5.97 | 73.9 | -0.379 | E.R. |
| *RsAHL30* | GWHPCBIT016616 | Chr4:58793643-58795110 | 912 | 303 | 31.75 | 5.89 | 63.1 | -0.545 | chlo |
| *RsAHL31* | GWHPCBIT016920 | Chr4:61408372-61410864 | 1023 | 340 | 35.55 | 9.21 | 63.97 | -0.479 | chlo |
| *RsAHL32* | GWHPCBIT017251 | Chr5:1588323-1589267 | 945 | 314 | 32.26 | 5.83 | 68.69 | -0.457 | E.R. |
| *RsAHL33* | GWHPCBIT020143 | Chr5:35528705-35529859 | 894 | 297 | 31.05 | 5.42 | 59.46 | -0.594 | chlo |
| *RsAHL34* | GWHPCBIT020532 | Chr5:37738401-37740520 | 1038 | 345 | 36.19 | 9.21 | 61.04 | -0.502 | nucl |
| *RsAHL35* | GWHPCBIT021610 | Chr5:44010561-44013196 | 1236 | 411 | 43.05 | 8.91 | 56.96 | -0.54 | chlo |
| *RsAHL36* | GWHPCBIT022881 | Chr6:967396-968325 | 930 | 309 | 31.83 | 5.98 | 68.58 | -0.418 | E.R. |
| *RsAHL37* | GWHPCBIT024390 | Chr6:11089807-11094174 | 2559 | 852 | 94.62 | 9.19 | 58.58 | -0.746 | nucl |
| *RsAHL38* | GWHPCBIT026050 | Chr6:41427899-41428914 | 903 | 300 | 30.87 | 6.67 | 63.67 | -0.589 | vacu |
| *RsAHL39* | GWHPCBIT026050 | Chr6:56123847-56124713 | 867 | 288 | 29.69 | 6.62 | 74.86 | -0.25 | nucl |
| *RsAHL40* | GWHPCBIT028736 | Chr6:59853361-59854311 | 951 | 316 | 33.48 | 6.59 | 67.56 | -0.607 | nucl |
| *RsAHL41* | GWHPCBIT030596 | Chr7:8574566-8575451 | 768 | 255 | 25.57 | 6.92 | 74.94 | -0.208 | nucl |
| *RsAHL42* | GWHPCBIT031187 | Chr7:13200906-13201793 | 885 | 294 | 30.43 | 6.66 | 63.61 | -0.558 | cyto |
| *RsAHL43* | GWHPCBIT031721 | Chr7:17531791-17532696 | 906 | 301 | 31.61 | 6.17 | 62.89 | -0.55 | nucl |
| *RsAHL44* | GWHPCBIT032919 | Chr8:3797015-3797959 | 945 | 314 | 32.29 | 6.7 | 62.99 | -0.601 | nucl |
| *RsAHL45* | GWHPCBIT034306 | Chr8:13172796-13174963 | 1347 | 448 | 46.82 | 9.46 | 50.36 | -0.846 | nucl |
| *RsAHL46* | GWHPCBIT036931 | Chr9:14367449-14370075 | 1302 | 433 | 45.95 | 5.62 | 59.01 | -0.6 | nucl |
| *RsAHL47* | GWHPCBIT037701 | Chr9:40000136-40001698 | 1116 | 371 | 39.62 | 9.16 | 54.5 | -0.656 | nucl |
| *RsAHL48* | GWHPCBIT038205 | Chr9:44990605-44996777 | 954 | 317 | 33.60 | 9.74 | 68.9 | -0.413 | nucl |
| *RsAHL49* | GWHPCBIT038490 | Chr9:47383735-47385586 | 1140 | 379 | 40.08 | 6.87 | 59.95 | -0.609 | nucl |
| *RsAHL50* | GWHPCBIT038650 | Chr9:48609182-48611665 | 1158 | 385 | 40.40 | 9.36 | 58.55 | -0.533 | nucl |
| *RsAHL51* | GWHPCBIT038760 | Chr9:49578918-49580964 | 1197 | 398 | 42.12 | 8.72 | 55.85 | -0.534 | chlo |
| *RsAHL52* | GWHPCBIT039386 | Chr9:53291501-53293252 | 1113 | 370 | 39.51 | 8.8 | 63.54 | -0.596 | nucl |

chlo: chloroplast; cysk: cytoskeleton; cyto: cytoplasm; E.R.: endoplasmic reticulum; extr: extracellular matrix; golg: golgi apparatus; mito: mitochondria; nucl: nucleus; plas: plasma membrane; pero: peroxisome; vacu: vacuole membrane.

**Supplementary Table 2.** Gene duplication of *RsAHL* genes in radish.

| Gene Name | Gene Name |
| --- | --- |
| *RsAHL4* | *RsAHL5*, *RsAHL38*, *RsAHL41*, *RsAHL42*, *RsAHL44* |
| *RsAHL6* | *RsAHL13*, *RsAHL17*, *RsAHL18*, *RsAHL46* |
| *RsAHL10* | *RsAHL21*, *RsAHL45*, *RsAHL52* |
| *RsAHL13* | *RsAHL17*, *RsAHL18*, *RsAHL46* |
| *RsAHL38* | *RsAHL41*, *RsAHL42*, *RsAHL44* |
| *RsAHL5* | *RsAHL42*, *RsAHL41* |
| *RsAHL7* | *RsAHL12*, *RsAHL19* |
| *RsAHL8* | *RsAHL11*, *RsAHL20* |
| *RsAHL12* | *RsAHL19*, *RsAHL40* |
| *RsAHL16* | *RsAHL26*, *RsAHL39* |
| *RsAHL17* | *RsAHL18*, *RsAHL46* |
| *RsAHL21* | *RsAHL45*, *RsAHL52* |
| *RsAHL24* | *RsAHL49*, *RsAHL47* |
| *RsAHL30* | *RsAHL33*, *RsAHL43* |
| *RsAHL35* | *RsAHL37*, *RsAHL51* |
| *RsAHL9* | *RsAHL22* |
| *RsAHL11* | *RsAHL20* |
| *RsAHL26* | *RsAHL39* |
| *RsAHL28* | *RsAHL40* |
| *RsAHL31* | *RsAHL34* |
| *RsAHL32* | *RsAHL36* |
| *RsAHL33* | *RsAHL43* |
| *RsAHL37* | *RsAHL51* |
| *RsAHL41* | *RsAHL42* |
| *RsAHL45* | *RsAHL52* |
| *RsAHL49* | *RsAHL50* |

**Supplementary Table 3.** Number and functions of cis-elements in radish *RsAHL* family.

| Name | Number | Functions |  |
| --- | --- | --- | --- |
| Box 4 | 191 | part of a conserved DNA module involved in light responsiveness |  |
| ARE | 119 | cis-acting regulatory element essential for the anaerobic induction |  |
| GT1-motif | 78 | light responsive element |  |
| G-box | 76 | cis-acting regulatory element involved in light responsiveness |  |
| ABRE | 72 | cis-acting element involved in the abscisic acid responsiveness |  |
| TCT-motif | 55 | part of a light responsive element |  |
| CGTCA-motif | 52 | cis-acting regulatory element involved in the MeJA-responsiveness |  |
| TGACG-motif | 52 | cis-acting regulatory element involved in the MeJA-responsiveness |  |
| TC-rich repeats | 41 | cis-acting element involved in defense and stress responsiveness |  |
| MBS | 38 | MYB binding site involved in drought-inducibility |  |
| GATA-motif | 30 | part of a light responsive element |  |
| I-box | 30 | part of a light responsive element |  |
| TGA-element | 30 | auxin-responsive element |  |
| LTR | 25 | cis-acting element involved in low-temperature responsiveness |  |
| AE-box | 24 | part of a module for light response |  |
| TCA-element | 21 | cis-acting element involved in salicylic acid responsiveness |  |
| MRE | | 20 | MYB binding site involved in light responsiveness |
| chs-CMA1a | 17 | part of a light responsive element |  |
| Sp1 | 13 | light responsive element |  |
| ATCT-motif | 12 | part of a conserved DNA module involved in light responsiveness |  |
| AT1-motif | 9 | part of a light responsive module |  |
| GA-motif | 9 | part of a light responsive element |  |
| TCCC-motif | 9 | part of a light responsive element |  |
| Box II | 8 | part of a light responsive element |  |
| ACE | 7 | cis-acting element involved in light responsiveness |  |
| ATC-motif | 7 | part of a conserved DNA module involved in light responsiveness |  |
| AuxRR-core | 7 | cis-acting regulatory element involved in auxin responsiveness |  |
| LAMP-element | 5 | part of a light responsive element |  |
| chs-CMA2a | 4 | part of a light responsive element |  |
| Gap-box | 4 | part of a light responsive element |  |
| GTGGC-motif | 2 | part of a light responsive element |  |
| chs-Unit 1 m1 | 2 | part of a light responsive element |  |
| 3-AF1 binding site | 1 | light responsive element |  |
| 4cl-CMA2b | 1 | light responsive element |  |
| ACA-motif | 1 | part of gapA in (gapA-CMA1) involved with light responsiveness |  |
| AuxRE | 1 | part of an auxin-responsive element |  |
| LS7 | 1 | part of a light responsive element |  |

**Supplementary Table 4.** The primer sequences were used in the study.

| Primer name | Forward sequence | Reverse sequence |
| --- | --- | --- |
| RsAHL1-qPCR | AAACAAACCGAAACCGCCC | GTAAGGGCTCATGGGAGGCT |
| RsAHL2-qPCR | GTTATCGGGTCCTATCTCC | TCCTCGTCCTCGTTGCTC |
| RsAHL3-qPCR | TGCAGCACAACCGTTCAA | CCGCTATCTCCACCATCAG |
| RsAHL4-qPCR | ATGGAAGGCGGCTACGAG | CCGACGGTTGTTGCTGTT |
| RsAHL5-qPCR | ATGGACGGTGGTTATGAT | GGTTGAGCCTGTTGTTGA |
| RsAHL6-qPCR | ATGGAGGAGAGAGAAGGAACGA | GATAGGGTTTAAGGACCGTGGT |
| RsAHL7-qPCR | ATGGATCCAGCCCAATCTCAT | TTGTTGATCGGGGTTTCTTTG |
| RsAHL8-qPCR | ATGGTTTTAGAGATGGAGTCAACCG | ACGGAGTTAGGAGATGGATTTGAG |
| RsAHL9-qPCR | CCATCAGGGTCTTGACTTAG | GGAGGTTTCGGTTTGTTT |
| RsAHL10-qPCR | CCGCCTGGGATGCTAATG | ATGTGGTGGAGGTGGGAC |
| RsAHL11-qPCR | TCAAGCGTTACACTTCGTC | TGTCATTCCACCTGTTCTG |
| RsAHL12-qPCR | CTCTCTCTCTCTGGCTCTTTTCTCC | ATCTCATCTTCCTCTAACGGAAGTC |
| RsAHL13-qPCR | CTCGCTGTGACTCTTTCCC | AGCGAGGTAGAGGG |
| RsAHL14-qPCR | GCGACACCGAGCCTCCTAT | CCGCTGAGAACGCAAACG |
| RsAHL15-qPCR | ATCTCAACAAGAGCCCCAGTTC | CAAGGACCTCTGCACCAATGTAT |
| RsAHL16-qPCR | ACCGACAACCACCACGAA | CCACCTAGTCCAGAGTCCA |
| RsAHL17-qPCR | ATGGAGGAGAGAGAAGGAACCA | CCCCACCGGAAAAAAGTTA |
| RsAHL18-qPCR | CCTATGGACCCACCACGAC | CGCATTCTCCACAGCCAC |
| RsAHL19-qPCR | ATGGACGGAACACAAAGCTCA | GTTGATGGTGGAGGAAGAACTC |
| RsAHL20-qPCR | GAACGAACCAGCAAGATCAGAA | ATCTGGTGAGAAGACGAAGTAGGA |
| RsAHL21-qPCR | GCTAACGGGCTCCTACAACC | GTAGGAGGAGGACCCAGAGAAA |
| RsAHL22-qPCR | CCGACGACGAAGACAACAAC | AACGTCTCCTCCTCCTCCG |
| RsAHL23-qPCR | ACCAGTGTATCTTATCAGGGTC | ACAAATGCTAAGGCCACC |
| RsAHL24-qPCR | TCCAGCAACAGCAGCATCC | GTTGCATAGTGGGAGACGAAGA |
| RsAHL25-qPCR | CCAATGCCATTCCCACTC | CCAATGCCATTCCCACTC |
| RsAHL26-qPCR | GCAGGTCGTCGGAGGTAA | CGTAAGCCACGTTCGTAAAA |
| RsAHL27-qPCR | ATGGCTGGAGGAAATATGGCT | GGTTGTTGTTGTTATTGGCTCTCT |
| RsAHL28-qPCR | AGCATCAGCAGCAACATAA | GAAGAGTGCTCGTTTGGAT |
| RsAHL29-qPCR | AACCTCCTCCAGCCATCA | GTCGTCTTCGTCGTCGTG |
| RsAHL30-qPCR | ATGGCGAACCCTTGGTGG | GTTGCTTCTGTGGTGCAAAGAC |
| RsAHL31-qPCR | AGACTCAACAGCCCTTTCA | CTTCCTGGGTTGTTCTGG |
| RsAHL32-qPCR | CCGCCTAGTTCATCTCAG | CGGTGGCTTTGGTTTGTTCT |
| RsAHL33-qPCR | AGAGTGGGAACGATGGAA | ACCCCAAAACACGTCTTG |
| RsAHL34-qPCR | AGCTATGGCGGGTTCTTA | CTTGGTTCAGGTTTGGGT |
| RsAHL35-qPCR | TCACAGTTAAGGGAGATGAAG | GTGGTGGGACTTACGAATT |
| RsAHL36-qPCR | ACACTCCGTCAACCATCAG | GAAAGAATCTCAAACCTCCCT |
| RsAHL37-qPCR | GGGTCATCACAGTCAAGGG | GGAATCACGGTGGTTGGA |
| RsAHL38-qPCR | GGAGGTAATGTCGGTGGTT | AGAGGTGGTCTTGAAGGTGTA |
| RsAHL39-qPCR | TCTAGGCACAACTTCTCGC | GTTTCCTCCAGCTCCACC |
| RsAHL40-qPCR | GCTCTCTCCCTCCACCTTTT | TTGTCTCGGTCGCGTTTTA |
| RsAHL41-qPCR | CACCAGATTCTGACCCGGT | CCAGAAGAGATTTCAAGAACATGA |
| RsAHL42-qPCR | ATGGACGGCGGTTACGAT | CAGGCTGATGTTGAGGTTGT |
| RsAHL43-qPCR | ATGGCGAATCCATGGTGG | GTTATTGCTGCTTCTGTGATGC |
| RsAHL44-qPCR | ATGGAAGGCGGTTACGAG | TTGCGGTTGAAGCTGTTG |
| RsAHL45-qPCR | TTCCTAATGGGCTCCTACAA | ATGCTGCTGCTGGTGATG |
| RsAHL46-qPCR | TGGAAGAGAGAGAAGGAACCAGCAA | TGGGTCCATATGGAAGACACCACCA |
| RsAHL47-qPCR | CTAATCTCCAGCAGCAGCATC | GTTGTTGTTGCATAGTGGGAGAC |
| RsAHL48-qPCR | GACAGAATTAGCTCTGGAGTGACTG | AGATGGTCTGGGAGCTATATGAAAT |
| RsAHL49-qPCR | ACTACTACCTCCAAAGAGGAGCCTT | ATGTTGTGTCCGAAGTCAGAGTG |
| RsAHL50-qPCR | CCAAAGAGGAGCCTTTACG | CGCTAGGGTTTAGCATCG |
| RsAHL51-qPCR | ATGTAACAATGAAAGTAATGCCGTA | TAGGTTGACGAAGTGTGACATTAGA |
| RsAHL52-qPCR | CGCCACTGGCAATGTTAA | TGGATCGGTTGGGATGTT |
| RsRPII-qPCR | ACAGGTTGTGGTGGTCGATC | CCTTCACTGCACCTGTGGAT |
| RsAHL14-OE | CTCGAG ATGAAAGGTGAAGGTAACGAGATGT | TCTAGA TCAGTAAGGCGGTGGAGCC |
| SlGA20ox-1-qPCR | CGAGAAGCCTTGTGCCTTAGTA | ATCAACTCCGTGATTCACCACA |
| SlGA20ox-2-qPCR | CCATCAAGACAACGTCTCAGGA | AGCGCCATAAATGTGTCACCTA |
| SlGA20ox-3-qPCR | AGAATATTGCAATGCCGTGAGC | AGTGGTTCTTGCTCACACCTAG |
| SlGA20ox-4-qPCR | ATCAAGATGATGTTGGTGGCCT | CCAATGTTGACCACAAAGGCAT |
| SlGA3ox-1-qPCR | TGTTGTCCTAACCCTTACCACG | GTTGGAAACCGGAGCAATTGAA |
| SlGA3ox-2-qPCR | TCTCCAAGTTCATGTGGCATGA | GTTAGGGTTAGGGCAACATGGA |
| SlGA3ox-3-qPCR | TGGCATGAAGGGTTCACCATTA | AGGGTTTGGGCAACATGGATAT |
| SlGA3ox-4-qPCR | GTCTTGCAGCACATACGGATTC | AATTTCGACAGGGGTGAGACTC |
| SlGA3ox-5-qPCR | TGGAAAAGCTAGCGGGAAGATT | GAATCCGTATGTGCAGCAAGAC |
| SlCPS-qPCR | GCTGCATTTCTTTGCGAATTCG | ATGGATGTCTCTGCCATGTGTT |
| SlKS-qPCR | GACCCAAACTCCCAGATGATGT | TGTCATTAAGAAGACGTCCGCA |
| SlKO-qPCR | CAGGAGGTTACTGGTTCAGACC | ACAACGGTGCAATAAAATGCCA |
| SlGA2ox-1-qPCR | GGTGGTGAATCATGGTGTCTCT | ACCATAGCCAAAAGGGTTAGCA |
| SlGA2ox-2-qPCR | GTTCTTCTCCTCTCCCCTCTCT | TAATCCCTCCGCCAACATTTCA |
| SlGA2ox-3-qPCR | TTTTGGAGGACCACCATTGAGT | TGTACAAGCTGTCTTCATCCCC |
| SlGA2ox-4-qPCR | GAACCTCATCGTTAATGCCTGC | CAGAGAGGGGAGAGGAGAAGAA |
| SlGA2ox-5-qPCR | TTCTTCTCCTCTCCCCTCTCTG | CAACCGATATCGCCACTTTGTC |

Note: red font indicates the cleavage site.
